# Emerging role of SENP1 in tumorigenesis and cancer therapy

**DOI:** 10.3389/fphar.2024.1354323

**Published:** 2024-02-08

**Authors:** Min Lin, Man Zhang, Bei Yi, Jinchi Chen, Siqi Wen, Ruiqi Chen, Tianyu Chen, Zhao Li

**Affiliations:** ^1^ Department of Experimental Research, Guangxi Medical University Cancer Hospital, Nanning, China; ^2^ Department of Gastrointestinal Surgery, Guangxi Clinical Research Center for Colorectal Cancer, Guangxi Medical University Cancer Hospital, Nanning, China

**Keywords:** SENP1, deSUMOylation, proliferation and apoptosis, invasion and metastasis, stemness, angiogenesis, metabolism, drug resistance

## Abstract

Acting as a cysteine protease, small ubiquitin-like modifier (SUMO)/sentrin-specific protease1 (SENP1) involved in multiple physiological and pathological processes through processing the precursor SUMO protein into mature form and deSUMOylating target protein. It has been reported that SENP1 is highly expressed and plays a carcinogenic role in various cancers. In this paper, we mainly explore the function and mechanism of SENP1 in tumor cell proliferation, apoptosis, invasion, metastasis, stemness, angiogenesis, metabolism and drug resistance. Furthermore, the research progress of SENP1 inhibitors for cancer treatment is introduced. This study aims to provide theoretical references for cancer therapy by targeting SENP1.

## Introduction

SUMO, discovered in 1996, is widely expressed in eukaryote to regulate target protein localization, activity and the interaction with other biomacromolecule through covalently modifying substrate proteins (Chang and Yeh, 2020). There are five different SUMO proteins encoding by human genome including SUMO1, SUMO2, SUMO3, SUMO4 and SUMO5. SUMO1, SUMO2 and SUMO3 are the main SUMO proteins while the expression of SUMO4 and SUMO5 is restricted to specific tissues (Kukkula et al., 2021). The amino acid sequence between SUMO2 and SUMO3 is 97% homologous, while they share only 50% homology with SUMO1 (Gareau and Lima, 2010). Since SUMO2 and SUMO3 cannot be distinguished by antibody. These two isoforms are collectively referred as SUMO2/3 (Hickey et al., 2012). Different amino acid sequence leads to that SUMO1 and SUMO2/3 modifies different substrates (Shen et al., 2006).

**FIGURE 1:**
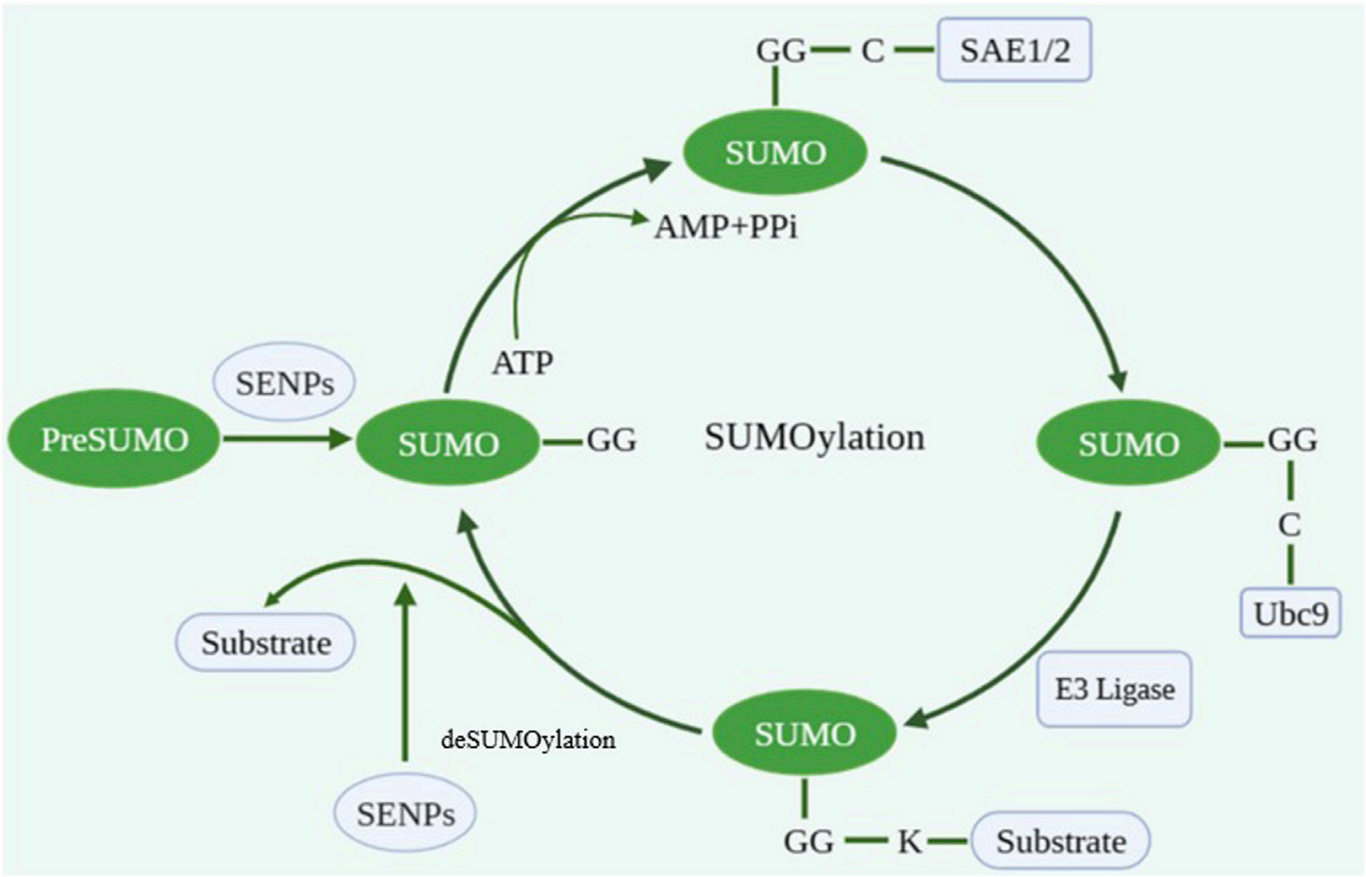
The process of SUMOylation. Created with BioRender.com.

As a crucial protein post-translational modification (PTM), SUMOylation participates in various biological processes including gene expression, DNA replication/repair, RNA processing, and nucleocytoplasmic transport. SUMOylation is a dynamic and reversible enzymatic cascade reaction process which is catalyzed by SUMO-specific activating enzyme (E1; SAE1 and SAE2), conjugating enzyme (E2; Ubc9) and ligating enzyme (E3) (Zhao, 2018). The SUMOylation process includes four phases: maturation, activation, conjugation and ligation (Ryu et al., 2020). The first step in the SUMO conjugation pathway is cleaving their COOH termini via hydrolyzing ATP to expose the diglycine (GG) residues required for conjugation. Second, the mature SUMO protein is activated by binding to activating enzyme E1. And then, the SUMO protein is transferred to conjugating enzyme E2. Finally, SUMO forms isopeptide bonds with the specific lysine residues (K) on substrate with the assistance of ligase E3 (Figure 1). The classic motif of SUMO modification site on target protein is ΨKXE (Ψ is an amino acid whose side chain is hydrophobic and X is any amino acid) (Tatham et al., 2001; Vertegaal, 2022).

The SUMOylation cycle is a dynamic and reversible process. The removal of SUMO protein from substrate protein is known as deSUMOylation, which is mediated by SUMO/sentrin-specific proteases (SENPs). The SENPs family is a class of cysteine proteases, including six members (SENP1, SENP2, SENP3, SENP5, SENP6, and SENP7) (Tokarz and Wozniak, 2021). They all have a conserved protease catalytic domain at the C-terminal while the N-terminal sequence and structure are different (Nayak and Müller, 2014). According to their sequence homology, cellular location and substrate specificity, they are divided into three subfamilies. SENP1 and SENP2, which are capable to deconjugate SUMO1 and SUMO2/3 modification, belong to the first subfamily while the second and the third subfamily of SENPs prefer SUMO2/3 as their substrates. SENP1 is the first mammalian enzyme to be reported and is primarily localized in the nucleus (Bailey and O'Hare, 2004). The homo sapiens SENP1 gene locates on chromosome 12q13.11 (Gong et al., 2000). SENP1 contains triad characteristics of cysteine proteases, consisting of Cys603, His533 and Asp550. It has been reported that SENP1 can process SUMO precursor into mature form and release SUMO protein from target protein. The mutation of the catalytic triad residues can abolish SENP1 functional activity (Liu W. et al., 2023). In recent years, overexpression of SENP1 has been reported in different types of cancers. Imbalance of target protein SUMOylation homeostasis mediated by SENP1 is closely associated with tumor development (Tokarz and Wozniak, 2021). The article will comprehensively summarize the regulatory mechanism of SENP1 and discuss the research progress of its inhibitors, aiming to provide new ideas for cancer therapy.

### The expression of SENP1 in cancers

SENP1 was overexpressed in breast cancer cell lines compared to immortalized mammary epithelia MCF10A cells. The expression of SENP1 in breast primary tumor tissues was significantly higher than that in normal tissues (Sun et al., 2018). Data from the Cancer Genome Atlas (TCGA) showed that SENP1 expression was significantly increased in breast tumors compared to adjacent normal tissues. SENP1 high signal is detected in the majority of triple-negative breast cancer (TNBC) clinical samples (Gao et al., 2022). Moreover, SENP1 expression in TNBC tissues is markedly higher than that in non-TNBC tissues (Wang Z. et al., 2016). For lung cancer, the mRNA and protein levels of SENP1 both are upregulated in cancer tissues (Wang RT. et al., 2013). In prostate cancer, SENP1 is overexpressed in prostatic intraepithelial neoplasia and prostate cancer lesion compared to normal prostate epithelia (Bawa-Khalfe et al., 2007). Another research also detected the overexpression of SENP1 in prostate intraepithelial neoplasia lesions and prostate cancer tissues from patients (Wang Q. et al., 2013). For liver cancer, the expression level of SENP1 is obviously higher in tumor tissues than para-carcinoma tissues (Tao et al., 2020). In pancreatic cancer, the SENP1 marker is expressed at high level (Ma et al., 2014). It was also observed that SENP1 was upregulated in colorectal cancer cell lines and clinical samples (Xu et al., 2011; Chen et al., 2021). The integration of GSE12452 and GSE53819 datasets identified eleven SUMOylation regulators whose mRNA expression upregulated in nasopharyngeal carcinoma, including SENP1. SENP1 also expresses highly in hematologic neoplasms. Overexpressed SENP1 is found in leukemia, multiple myeloma (MM) and mantle cell lymphoma (Ohbayashi et al., 2008; Xu et al., 2015; Wang FF. et al., 2016; Zhang et al., 2021; Niu et al., 2022; Liu J. et al., 2023). In conclusion, SENP1 might play an oncogenic role not only in solid tumor, but also in malignant tumor, which deserves further exploring about the specific mechanism.

### SENP1 in tumor cell proliferation

Cancer is characterized by the process of normal cell evolving into tumor cell to acquire special abilities, including the ability of tumor cells to proliferate indefinitely due to the severe dysregulation of cell cycle. Therefore, studying the mechanism of unlimited proliferation of tumor cells will help to find new treatment options and strategies. In nasopharyngeal carcinoma, SENP1 overexpression enhances cell viability, cell proliferation rate and cell clonality (Zhang et al., 2023). A study of Wilms tumor showed that overexpressed SENP1 dramatically increased cell proliferate capacity through stimulating Cyclin E1 expression. SENP1 silence diminished the expression of proliferation marker Ki67 and proliferating cell nuclear antigen (PCNA). Flow cytometry proved that SENP1 silence promoted G0/G1 phase arrest and decreased the S phase cell proportion (Zhu et al., 2021). In mantle cell lymphoma, SENP1 inhibits cell proliferation while has no significant effect on cell cycle distribution (Zhang et al., 2021). MicroRNA-186 overexpression directly downregulates SENP1 protein expression and inhibits cell proliferation in renal cell carcinoma (Jiao et al., 2018). The study in hepatocellular carcinoma demonstrated that SENP1 deletion inhibited proliferation and induced cell cycle dysregulation through the deSUMOylation of ubiquitin conjugating enzyme E2 T (UBE2T) (Tao et al., 2020). Overexpressed SENP1 promotes androgen receptor-dependent cell proliferation in prostate cancer cells (Bawa-Khalfe et al., 2007). Knockdown SENP1 significantly attenuates colony formation ability in prostate cancer (Wang Q. et al., 2013). Additionally, microRNA-145 mediates proliferation arrest through suppressing SENP1 activity in prostate cancer (Wang C. et al., 2015). In colorectal cancer, microRNA-133α-3p targets SENP1 to block cell cycle (Zhou GQ. et al., 2018). SENP1 silence upregulates cyclin dependent kinase inhibitors such as p16, p19, p21 and p27, resulting in colorectal cancer cell cycle arrest (Xu et al., 2011). In summary, these results illustrate that SENP1 promotes tumor cell proliferation through various mechanisms.

### SENP1 in tumor cell apoptosis

Apoptosis, considered as an important mechanism to prevent tumor progression is an autonomous and orderly form of cell death controlled by specific genes. The resistance of tumor cells to apoptosis is a significant factor in cancer treatment failure (Schmitt, 2003; Hanahan and Weinberg, 2011). Downregulation of SENP1 increases the apoptosis rate of human glioma cells (Zhang QS. et al., 2016). Another research of astroglioma cells indicated that SENP1 knockdown downregulated anti-apoptotic protein B-cell lymphoma-2 (Bcl-2) through blocking nuclear factor kappa-B (NF-кB) signal activation, which promotes cell apoptosis (Xia et al., 2016). Similarly, SENP1 deletion inhibits NF-кB signaling pathway, resulting in tumor cell apoptosis in multiple myeloma (Xu et al., 2015). In osteosarcoma, overexpressed SENP1 decreases the expression of apoptotic protein BCL2-Associated X (BAX) while upregulates Bcl-2, consequently preventing cell apoptosis (Wang et al., 2018). In mantle cell lymphoma (MCL), SENP1 knockdown increases apoptosis by suppressing JAK-STAT5 signal transduction and increasing the expression of tumor suppressor cytokine signaling 2 (SOCS2) (Zhang et al., 2021). Conclusively, these studies suggest that SENP1 leads to apoptosis resistant and promotes cell survival.

### SENP1 in tumor invasion and metastasis

Tumor invasion and metastasis are key factors leading to poor prognosis and death of cancer patients. Deeply exploring the specific regulatory mechanism of SENP1 in tumor invasion and metastasis is helpful to limit tumor progression. The biological process by which epithelial cells transform into cells with a mesenchymal phenotype is called epithelial-mesenchymal transition (EMT), which is considered as an important pathway for tumor invasion and metastasis (Pastushenko and Blanpain, 2019). EMT is characterized by downregulation of epithelial markers (cytokeratin and E-cadherin) and upregulation of mesenchymal markers (N-cadherin, vimentin, and fibronectin) (Mittal, 2018). SENP1 has been reported to promote tumor invasion and metastasis in various cancers. In breast cancer, SENP1 inhibition prevents the deSUMOylation of hypoxia-inducible factor 1α (HIF-1α), ultimately leading to HIF-1α degradation and cancer metastasis suppression (Jia et al., 2020). SENP1 directly contributes to the bone metastasis of prostate cancer cells. Mechanistically, SENP1 increases the expression of bone remodeling proteins such as matrix metalloproteinases 2 (MMP2) and matrix metalloproteinases 9 (MMP9) through activating HIF-1α signaling pathway (Wang Q. et al., 2013). Matrix metalloproteinases (MMPs) can facilitate the degradation of basement membrane and promote cell invasion. Studies in neuroblastoma demonstrated that SENP1 silence suppressed tumor invasion and metastasis via downregulating MMP2 and MMP9 levels (Xiang-Ming et al., 2016). The function of SENP1 in invasion and metastasis was also proved in several other cancers including TNBC and pancreatic cancer (Ma et al., 2014; Wang Z. et al., 2016). In TNBC, SENP1 deSUMOylates GATA binding protein 1 (GATA1) at lysine residue K137 and promotes the binding of GATA1 and COP9 signalosome complex subunit 5 (CSN5) promoters. CSN5 is a deubiquitinate ligase of Zinc finger E-box-binding homeobox 1 (ZEB1). Thus, activated CSN5 prevents ZEB1 degradation and maintains its protein stability, resulting in EMT and tumor metastasis (Gao et al., 2022). In prostate cancer, SENP1 silence enhances E-cadherin expression while inhibits vimentin expression (Zhang et al., 2017). In hepatocellular carcinoma, SENP1 knockdown suppresses EMT by increasing the expression of E-cadherin and zonula occludens-1 (ZO-1) while decreasing fibronectin and N-cadherin (Zhang W. et al., 2016). Researchers also found that SENP1 knockdown suppressed the invasive ability of osteosarcoma cells through modulating EMT marked genes (Wang et al., 2018). In nasopharyngeal carcinoma, SENP1 increases STAT1 protein level and promotes its nuclear translocation by inhibiting STAT1 SUMOylation, resulting in cancer invasion and metastasis (Zhang et al., 2023). Additionally, SENP1 facilitates the lung metastasis of colorectal cancer cells (Zhou et al., 2021). For upstream of SENP1, microRNA-1236 abolishes hypoxia-induced EMT through suppressing SENP1 (Chen et al., 2016). Summarily, SENP1 promotes cancer invasion and metastasis mainly through regulating EMT as well as other signaling pathway.

### SENP1 in tumor stemness

Cancer stem cells (CSCS) are a group of tumor cells with the ability of self-renewal, which is a crucial factor of cell carcinogenesis and cancer progression. Multiple studies have identified that SENP1 plays an important role in cancer stemness. In hepatocellular carcinoma cells, SENP1 induces stemness-related genes expression such as Oct3/4, Nanog, NOTCH1 and BMI-1 through enhancing the stability and transcriptional activity of HIF-1α, ultimately leading to cancer cell self-renewal (Cui et al., 2017). In renal cell carcinoma cells, overexpressed SENP1 enhances the transcriptional activity of hypoxia-inducible factor 2α (HIF-2α) through deSUMOylation, resulting in stemness-related genes expression increasing (Lee et al., 2022). SENP1 increases the sphere formation ability of hepatocellular carcinoma cells through deSUMOylating HIF-1α (Sun et al., 2023). CD24 is a known marker of liver cancer stem cells. SENP1 overexpression increases CD24^+^ cell population and upregulates stemness-related genes expression in liver cancer cells (Dai et al., 2023). In glioblastoma, the study observed that SENP1-mediated deSUMOylation of methyltransferase like 3 (METTL3) promoted MYC protein expression, thereby accelerating self-renewal of tumor cells (You et al., 2022). A recent study showed that ubiquitin-specific protease 51 (USP51) could directly bind to Elongin C (ELOC) and form a functional complex with VHL E3 ligase to suppress ubiquitin-dependent proteasomal degradation of HIF-1α. The deSUMOylation of ELOC induced by SENP1 promoted USP51 bind to ELOC and facilitated the deubiquitylation and stabilization of HIF-1α, consequently enhancing colorectal cancer cells stemness (Mu et al., 2023). In summary, SENP1 plays an important role in tumor stemness and malignant development. Targeting SENP1 might be a potential therapeutic target.

### SENP1 in tumor angiogenesis

Tumor angiogenesis is a hallmark of malignant transformation of cancer (Hanahan, 2022). Tumor growth is largely dependent on growth of blood vessels and formation of new blood vessels to provide nutrients and oxygen for tumor metabolism as well as remove metabolism waste (Hanahan and Weinberg, 2011). Vascular endothelial growth factors (VEGFs) are critical regulators that stimulate endothelial cells to proliferation, migration, and forming new vessels (Viallard and Larrivée, 2017). SENP1-deficient endothelial cells show increased SUMOylation of VEGFR2 through which impaired VEGFR2 signaling pathway. Mechanistically, SUMOylation retains VEGFR2 in Golgi and reduces its membrane surface distribution, thereby reducing angiogenesis in endothelial cells. However, VEGFR2 will be deconjugated rapidly and transported to plasma membrane for strong angiogenesis response when SENP1 expression exists (Zhou HJ. et al., 2018). In addition, SENP1 can increase the expression and secretion of VEGFs under hypoxic condition (Xu et al., 2010; Wang et al., 2018; Zhou and Sun, 2020). Similarly, SENP1 allows HIF-1α to escape degradation and maintain stability, subsequently facilitating the transcription of downstream gene VEGF under hypoxia through which further induces angiogenesis of adjacent glomerular endothelial cells via binding VEGFR2 (Wang L. et al., 2015). Furthermore, overexpression SENP1 in a mouse model enhances HIF-1α protein stability, which increases VEGF production and angiogenesis (Bawa-Khalfe et al., 2010). Fibroblast growth factor (FGF2) is recognized as the first discovered proangiogenic molecule, which facilitates angiogenesis through activating FGF receptor 1 (FGFR1) signal in endothelial cells (Lee et al., 2023). Researchers demonstrated that SENP1-induced deSUMOylation of FGFR1 acted as a crucial mechanism in response to proangiogenic stimuli (Zhu et al., 2022). NOTCH pathway is a prominent negative regulator of endothelial sprouting and vascular growth (Benedito et al., 2009). SENP1 deletion promotes NOTCH1 to cleavage and stabilize NOTCH1 intracellular domain (N1ICD) for translocation to nucleus through enhancing the SUMOylation of NOTCH1, consequently activating NOTCH signal and resulting in less angiogenesis (Zhu et al., 2017). In conclusion, SENP1 plays an important role in endothelia angiogenesis through various pathway. However, the direct evidence of SENP1 function in tumor angiogenesis requires further investigation.

### SENP1 in tumor metabolism

Tumor growth mainly obtains energy through glycolytic pathway. Studies revealed that SENP1 played an essential role in Warburg effect. SENP1 and HIF-1α interacts with each other to regulate tumorigenesis through enhancing glycolysis in prostatic carcinoma cells (Wang et al., 2019). Another research also implicated that SENP1 contributed to tumor metabolism in prostate cancer cells. Hexokinase 2 (HK2) is a crucial molecule that activates the glycolysis pathway of tumor cells. The study found that SENP1 induced HK2 deSUMOylation and facilitated HK2 to combine with mitochondria. This process greatly consumes glucose and produces lactic acid, thereby supporting cancer cell proliferation (Shangguan et al., 2021). In renal clear cell carcinoma, there is a positive connection between SENP1 and glycolysis level. SENP1 enhances HIF-1α protein stability, which further promotes the expression of key glycolysis enzymes and increases glycolysis flux (Dong et al., 2016). Conclusively, SENP1 participates in tumor metabolism mainly through regulating glycolytic pathway.

### SENP1 in tumor drug resistance

At present, drug resistance is a great challenge affecting therapy efficacy and prognosis of cancer patients. SENP1 has been reported as a promising therapeutic target to overcome drug resistance in a variety of cancers. In colorectal cancer, SENP1 drives chemotherapy resistance through reducing RNF168 SUMOylation. On the contrary, disrupting SENP1 enhances sensitivity to chemotherapeutic drugs (Wei et al., 2023). Overexpressed SENP1 facilitates JAK2 to accumulate in cytoplasm through deSUMOylating JAK2, thereby activating JAK2/STAT3 signaling transduction and leading to platinum therapy resistance (Li et al., 2021). The deSUMOylation of HK2 mediated by SENP1 desensitizes chemotherapy response in prostate cancer cells with docetaxel treatment (Shangguan et al., 2021). SENP1 activates sirtuin 3 (SIRT3) by preventing its proteasome degradation through which exacerbates resistance against chemotherapy in acute myeloid leukemia (AML) cells (Zhang et al., 2022). In MM cells, SENP1 is regarded as a key modifier of steroid receptor coactivator-3 (SRC-3) stability. SENP1-mediated SRC-3 deSUMOylation attenuates its K11-linked polyubiquitination and thus SENP1 knockdown accelerates the degradation of SRC-3 and remarkably overcomes resistance to proteasome inhibitors (Guo et al., 2022). Furthermore, SENP1 was identified as a desensitization factor for cisplatin treatment in ovarian cancer (Ao et al., 2015). Targeting SENP1 can significantly reduce irinotecan resistance in colorectal cancer patients (Chen et al., 2021). Non-small cell lung cancer (NSCLC) patients with higher SENP1 expression show lower rates of complete response, higher partial and non-response rate to chemoradiotherapy (Liu et al., 2018). The expression of SENP1 is dramatically increases in multidrug-resistant hepatocellular carcinoma cells (Qin et al., 2014). MicroRNA-122 targets SENP1 to decrease drug-resistance protein levels (p-glycoprotein and multidrug resistance protein), thereby attenuating chemoresistance (Dai et al., 2023). In summary, SENP1 promotes drug resistance in various cancers through different molecule mechanism.

### The prognostic value of SENP1 in cancer

Many researches have showed that SENP1 has prognostic value in various cancers. In Wilms tumor, the overall survival (OS) and disease-free survival (DFS) of patients with high SENP1 expression are dramatically shorter than those with low SENP1 expression (Zhu et al., 2021). High level of SENP1 predicts poor prognosis in prostate cancer patients (Shangguan et al., 2021). The positive expression of SENP1 in prostate cancer patients is significantly associated with poor survival (Li et al., 2013). Plasma exosome-derived SENP1 is a potential prognostic biomarker in osteosarcoma. Patients with higher SENP1 content have worse prognosis and lower survival rate (Wang et al., 2021). Similarly, melanoma patients with high plasma exosome-derived SENP1 level have shorter OS and DFS. SENP1 high expression represents larger tumor volume, deeper invasion extent, later pathological stage, and distant metastasis (Hu et al., 2021). In bladder cancer, the level of SENP1 in urine can detect tumor recurrence (Brems-Eskildsen et al., 2010). In AML, SENP1 decreases after induction therapy and its reduction predicts low disease risk, favorable treatment response, and long survival (Liu J. et al., 2023). Multivariate Cox regression analysis showed that SENP1 was an independent prognostic factors for the survival of NSCLC patients (Mu et al., 2014). Also, SENP1 is able to be used as a prognostic biomarker for glioblastoma (Li and Meng, 2021). In addition, high expression of SENP1 was strongly related to poor prognosis in renal cell carcinoma, nasopharyngeal carcinoma and colorectal cancer (Lee et al., 2022; Wei et al., 2023; Zhang et al., 2023). Collectively, these findings clarify that SENP1 is of great value in cancers prognosis.

### Inhibitors of SENP1

**FIGURE 2:**
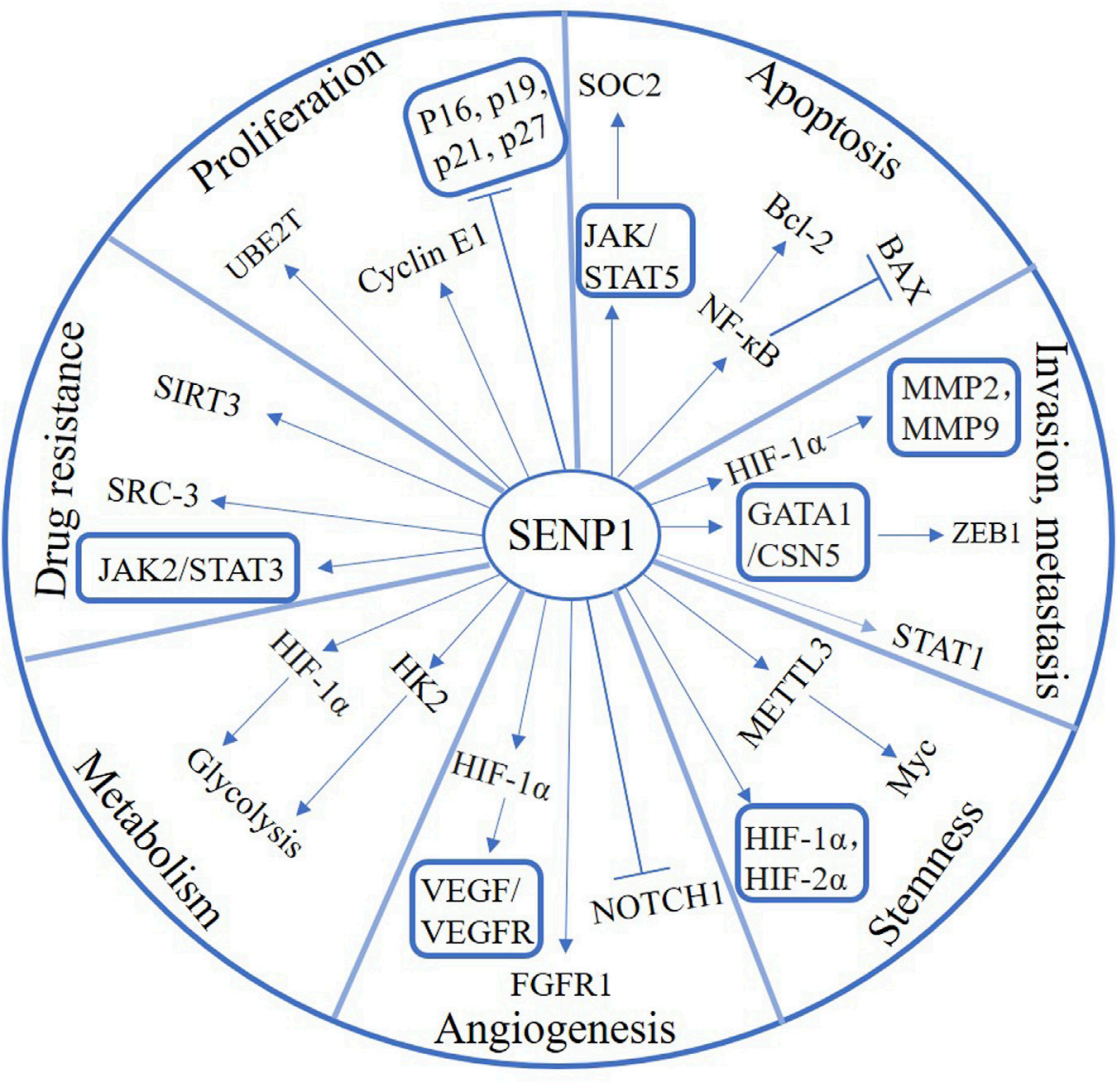
The function and signaling pathway of SENP1 in various cancers.

SENP1 plays a crucial role in tumor progression and is expected to be a critical target for cancer therapy (Figure 2). Therefore, the development of SENP1 inhibitors might bring new hope for cancer treatment. In recent years, tremendous efforts have been spent in SENP1 inhibitors study and development. Next, we will summarize the progress of SENP1 inhibitors in this paper, aiming to explore beneficial treatments for cancers (Table 1).

**TABLE 1:** SENP1 inhibitors.

Inhibitor	Name/Source	Type	IC50 (μM)	References
**Momordin Ιc**	Pentacyclic triterpenoid	Natural	15.37	(Li et al., 2021; Wu et al., 2016; Xianjun et al., 2021)
**Triptolide**	Chinese herb Tripterygium wilfordii Hook F	Natural	0.0203 (PC-3)	Huang et al. (2012)
			0.009754 (LNCaP)	
**Hinokiflavone**	Plant flavonoid family	Natural	/	Pawellek et al. (2017)
**Bethanidine**	Adrenergic neuron blocking factor	Clinical drug	/	Taghvaei et al. (2022)
**Vialinin A**	Chinese mushroom Thelephora vialis	Natural	1.64 ± 0.23 (rhSENP1)	Yoshioka et al. (2016)
			1.89 ± 0.04 (cSENP1)	
**Thelephantin G**			2.48 ± 0.02 (rhSENP1)	
			1.89 ± 0.04 (cSENP1)	
**Streptonigrin**	*Streptomyces* flocculus	Natural	0.518 ± 0.100 (SENP1)	Ambaye et al. (2018)
			6.919 ± 0.676 (SENP2)	
			5.205 ± 0.853 (SENP6)	
**Gallic acid**	Endogenous plant polyphenol	Natural	/	Taghvaei et al. (2021)
**Topotecan**	Pentacyclic monoterpene alkaloid	Clinical drug, Synthetic	/	Niu et al. (2022)
**7E-CPT**				
**Ursolic acid**	Pentacyclic triterpene	Natural	2.58	Wei et al. (2022a)
**Compound 1**	Ursolic acid	Synthetic, Selective-inhibitor	0.51	Wei et al. (2022b)
**Compound 2**	Benzodiazepine	Synthetic, Selective-inhibitor	15.5	Qiao et al. (2011)
			13.0 (PC-3)	
**Compound 3**			9.2	
			35.7 (PC-3)	
**Compound 4**	4′-methoxy-biphenyl-3-carboxylic acid	Synthetic, Selective-inhibitor	3.5	Zhao et al. (2016)
	3-(3-Phenyl-propionylamino)-benzylamide			
**Compound 5**	Benzothiophene-2-carboxamide	Synthetic, Pan-inhibitor	1.3 (SENP1)	Wang et al. (2020)
			0.7 (SENP2)	
			22.7 (SENP5)	
**GN6860**	1-[4-(N-benzylamino)phenyl]-3-phenylurea	Synthetic, Selective-inhibitor	39.5 ± 0.8	Uno et al. (2012)
**GN6958**			29.6 ± 0.5	
**Compound 6**	2-(4-Chlorophenyl)-2-oxoethyl 4-benzamidobenzoat	Synthetic, Selective-inhibitor	2.4	Chen et al. (2012)
**Compound 7**			1.080 ± 0.010	
**Compound 8**			1.856 ± 0.205	
**Compound 9**			1.735 ± 0.020	
**Compound 10**			1.175 ± 0.033	
**SPI-01**	Sulfonyl-benzene	Synthetic, Pan-inhibitor	5.9 ± 1.4 (SENP1)	Madu et al. (2013)
			2.9 ± 1.6 (SENP2)	
			3.5 ± 1.5 (SENP7)	
**Compound 11**	Pyridone scaffold	Synthetic, Selective-inhibitor	22 (0.1% BSA)	Lindenmann et al. (2020)
			3.7 (0.01% CHAPS)	
**Compound 12**			20 (0.1% BSA)	
			0.99 (0.01% CHAPS)	
**Compound 13**			51 (0.1% BSA)	
			7.5 (0.01% CHAPS)	

Momordin Ιc (Mc), a natural pentacyclic triterpenoid, presents a good inhibitory activity of SENP1 with an IC50 value of 15.37 μM. The cellular thermal shift assay (CETSA) and the drug affinity responsive target stability (DARTS) assay show that Mc directly interacts with SENP1 and inhibits its activity. Mc inhibits proliferation and promotes apoptosis in prostate cancer cells. However, SENP1 overexpression reversals Mc-induced proliferation inhibition and apoptosis. In nude mouse xenograft model, Mc significantly attenuates tumor growth (Wu et al., 2016). Furthermore, Mc treatment dramatically overcomes platinum-resistance in ovarian cancer (Li et al., 2021). In colorectal cancer, Mc induces cell cycle arrest and apoptosis through inhibiting SENP1-mediated c-Myc deSUMOylation (Xianjun et al., 2021).

Triptolide, an active component extracted from the Chinese herb Tripterygium wilfordii Hook F, has been reported to exhibit antitumor effects in various cancers. In prostate cancer, Triptolide decreases SENP1 mRNA and protein levels in both dose-dependent and time-dependent manner, resulting in cellular SUMOylation level increasing. *In vitro*, triptolide inhibits cell proliferation and induces cell apoptosis. And *in vivo*, triptolide decreases the weight and volume of xenograft tumors, which indicates that Triptolide has potent anti-tumor effect through suppressing SENP1 (Huang et al., 2012).

Hinokiflavone belongs to a plant flavonoid family, which has antitumor effects. Studies found that Hinokiflavone induced cell cycle arrest and cell death in both cervical cancer and acute promyelocytic leukemia. Hinokiflavone can inhibit target protein deSUMOylation through directly binding to SENP1 and inhibiting its activity (Pawellek et al., 2017).

Bethanidine, a strong adrenergic neuron blocking factor, is commonly used to treat heart failure in clinical practice. Through performing molecular docking and molecular dynamics simulation of Bethanidine with SENP1, researchers found that Betanidine constituted a stable complex with SENP1 to inhibit its activity. Although the specific mechanism needs to be further studied, these data provide the possibility of Bethanidine targeting SENP1 for cancer therapy (Taghvaei et al., 2022).

Vialinin A and thelephantin G are para-terphenyl compounds isolated from the edible Chinese mushroom Thelephora vialis. Both of them can inhibit the enzymatic activity of SENP1. Vialinin A and thelephantin G inhibit full-length recombinant human SENP1 (rhSENP1) activity with IC50 values of 1.64 ± 0.23 μM and 2.48 ± 0.02 μM. The IC50 values of Vialinin A and thelephantin G inhibit catalytic domain human SENP1 (cSENP1) are 1.89 ± 0.04 μM and 1.52 ± 0.06 μM, respectively. The structure-activity relationship (SAR) implicates that two benzyl acetyl moieties and two phenyl acetyl moieties of vialinin A and thelephantin G play an important role in inhibiting SENP1 activity. Moreover, the inhibition of SENP1 activity requires the 2-hydroxymoiety of p-terphenyl skeleton. The catechol moiety of center benzyl ring of p-terphenyl skeleton is also critical (Yoshioka et al., 2016).

Streptonigrin (SN) is a natural product isolated from *Streptomyces* flocculus. Recently, SN was identified as a small molecule inhibitor of SENP1. The study tested the SENPs−SUMO inhibition potency of SN by using different combinations of SENPs and AMC-tagged fluorogenic SUMO substrates. When SUMO1 acted as a substrate, SN preferred to inhibit SENP1 activity (IC50 = 0.518 ± 0.100 μM) rather than SENP2 activity (IC50 = 6.919 ± 0.676 μM). In order to investigate the specificity of SN, they tested SENP6 activity through using SUMO2−AMC as a substrate. The result proved that the inhibitory effect of SN on SENP6 was similar to that on SENP2 (IC50 = 5.205 ± 0.853 μM). These results indicate that SN has higher SENP1 inhibitory effect compared with other SENPs (Ambaye et al., 2018).

Gallic acid (GA), an endogenous plant polyphenol, exists abundantly in tea and fruits. This compound exerts antitumor effects through regulating cell cycle, cell apoptosis, angiogenesis, invasion and metastasis. By performing molecular docking and molecular dynamics simulation, studies found that GA suppressed SENP1 catalytic activity through directly interacting with its active site. GA shows high stability, high hydrogen bonds, high binding energy and the highest intermolecular bonds with SENP1. Moreover, Gallic acid has lower toxicity than Mc, indicating that Gallic acid is an ideal SENP1 inhibitor in cancer treatment. However, these data were obtained by computational analysis and still required experimental verification (Taghvaei et al., 2021).

Camptothecin is a pentacyclic monoterpene alkaloid isolated from the Chinese tree Camptotheca acuminate. Topotecan and irinotecan, two camptothecin derivatives, are currently used as anti-tumor drugs in clinic. Topotecan is mainly used in ovarian and lung cancer. And Irinotecan is commonly used in colorectal cancer therapy. In acute lymphoblastic leukemia (ALL), Topotecan was found to inhibit cells proliferation through SENP1 reduction. 7-ethylcamptothecin (7E-CPT), another camptothecin derivative, can downregulate SENP1 mRNA and protein levels, which suppresses ALL cells proliferation and induced apoptosis (Niu et al., 2022).

Ursolic acid (UA) is a natural pentacyclic triterpene which has been identified as an effective SENP1 inhibitor with an IC50 value of 2.58 μM. One study examined the SENP1 inhibitory efficiency of 29 commercially available natural ursane-type aglycones. Pomolic acid and tormentic acid are identified as effective SENP1 inhibitors with IC50 values of 5.1 μM and 4.3 μM. They can reverse cisplatin resistance greatly in ovarian cancer cells. Compared with cisplatin (IC50 = 28.23 μM) only, the IC50 values of cisplatin decreased to 3.69 μM (with pomolic acid) and 2.40 μM (with tormentic acid), which indicates the synergy effect between cisplatin and pomolic acid or tormentic acid (Wei et al., 2022a). However, some medicinal defects such as poor water solubility, rapid metabolism, and low bioavailability limit their clinical application. Another research designed a series of pentacyclic triterpene derivatives based on the structure of UA. Introducing hydrophilic or basic side-chain moieties is beneficial to improve the pharmaceutical properties of UA, and it is a rational drug design strategy in structure modifications based on pentacyclic triterpenes. The molecular docking experiment showed that the 3-hydroxyl group was the key structure for the interaction between UA and SENP1, and the 28-carboxyl group pointed to the solvent region. Therefore, they retained the 3-hydroxyl group and modified the 28-carboxyl group with hydrophilic amide to synthesize compound 1 which could inhibit SENP1 in a dose-dependent manner with an IC50 value of 0.51 μM. Researchers also demonstrated that UA and its derivatives could increase the radiosensitivity of HeLa cells. There is a dramatic positive correlation between SENP1 inhibitory activity and the sensitivity enhancement ratio (SER). Compound 1 presents the best radiosensitive activity with a SER value of 1.45. This is the first time to develop small molecule SENP1 inhibitors as radiosensitizers, which is of great significance for its application in tumor radiotherapy (Wei et al., 2022b).

Another research reported the design, synthesis and biological evaluation of SENP1 inhibitors based on benzodiazepines. In the crystal structure of SENP1 complexed with immature SUMO1, the catalytic Cys603 is located in a cleft. Once substrate binds, the cleft will be closed to form a channel-like structure. The cleft is occupied by the terminal peptide tail of precursor SUMO, leading to numerous hydrogen bonds formation. They observed that the benzodiazepine core structure could attach into the catalytic cleft and mimic the conformation of substrate peptides. The formyl group binds Cys603 and forms a covalent bond. Also, the substitution at the C–NH2 position might have an orientation similar to that of substrate peptides. Thus, they synthesized a series of carboxamides with extended substituents on the phenyl. Compound 2 was synthesized by installing NHCbz at the C (4′) position of the phenyl. They found its activity improved obviously with an IC50 value of 15.5 μM. The research further synthesized compound 3 through transferring the NHCbz group to the C (3’) position. Compound 3 showed higher activity (IC50 = 9.2 μM). In order to evaluate the inhibitory effect of compound 3, they subjected it to the SUMO-ΔRanGAP cleavage assay and found that compound 3 attenuated the enzymatic hydrolysis of SUMO-ΔRanGAP through SENP1 inhibition. In addition, they detected the inhibitory effect of them in prostate cancer cells. The IC50 values of compound 2 and compound 3 against prostate cancer cells were calculated to be 13.0 μM and 35.7 μM. This is the first report about designing and synthesizing SENP1 inhibitors, which opens the way for further research of SENP1 inhibitors based on benzodiazepines (Qiao et al., 2011).

Studies have identified eleven SENP1 inhibitors with various scaffolds through silico screening. Based on their structure, a series of new SENP1 inhibitors were designed and synthesized. Compound 4 (IC50 = 3.5 μM) has the highest SENP1 inhibitory effect (Zhao et al., 2016). They also developed many benzothiophene-2-carboxamide inhibitors based on protein structures of SENP1, SENP2, and SENP5. Compound 5 presented the best inhibitory potency with IC50 values of 1.3 μM (SENP1), 0.7 μM (SENP2), and 22.7 μM (SENP5) (Wang et al., 2020).

Masaharu et al. developed 1-[4-(N-benzylamino)phenyl]-3-phenylurea derivatives based on the HIF-1α inhibitor (GN6797) as SENP1 inhibitors. GN6797 was found to interact SENP1 directly by pull-down assay. In order to detect the effect on SENP1 enzyme activity, they combined GN6767 with SENP1 catalytic domain and incubated with fluorogenic SUMO-1-AMC (7-amino-4-methylcoumarin). GN6767 showed 40% inhibition of SENP1 endopeptidase activity at 100 μM concentration. However, this inhibition disappeared at 50 μM concentration. They found that the methyl substituent could increase the inhibitory potency of SENP1. GN6860 and GN6958 inhibit SENP1 protease activity in a concentration-dependent manner. GN6860 showed 74% inhibition of SENP1 endopeptidase activity at 50 μM concentration. GN6958 exhibited 97% inhibition at the same concentration. Their IC50 values are 39.5 ± 0.8 μM and 29.6 ± 0.5 μM, respectively. The study further demonstrated that GN6958 displayed no significant inhibition toward other proteases in the range of 10–100 μM concentrations such as trypsin, chymotripsin and thermolysin. These results suggest that GN6958 is a selective inhibitor of SENP1 enzymatic activity (Uno et al., 2012).

Chen et al. screened 38 compounds from SPECS library by using virtual screening and docking methods. The study identified compound 6 as the highest inhibitory potency of SENP1 with an IC50 value of 2.4 μM. Compound 6 can embed well in the binding site composed with Trp465, Asp468, Phe496, His529, Val532, Gln597 and Cys603. The amide nitrogen and carbonyl oxygen of it bonds with the side chain nitrogen of Gln597 and epsilon 2 nitrogen of His529, respectively, which stabilizes its extended binding, resulting in SENP1 inhibition. Thus, the research further designed and synthesized a series of 2-(4-Chlorophenyl)-2-oxoethyl 4-benzamidobenzoate derivatives based on compound 6. Considering the perpendicular π-π interaction between meta benzene-ring substitute and the phenyl ring of Phe496, they designed compound 7 through replacing the meta methoxy with a benzoxy, which showed efficient inhibition effect of SENP1 (IC50 = 1.080 ± 0.010 μM). Compound 8, 9, 10 were obtained by replacing the meta methoxy with a nitro, a fluorine and a bromine, respectively. These compounds all exhibited enhanced inhibitory potency of SNEP1 with IC50 values of 1.856 ± 0.205 μM, 1.735 ± 0.020 μM and 1.175 ± 0.033 μM, respectively (Chen et al., 2012).

Madu et al. screened 250,000 compound libraries and found the most potent compounds against SENP1 contained sulfonyl-benzene group. They found the inhibitory potency of these compounds depended on both enzyme and substrate. When SUMO-1 precursor acted as a substrate, four compounds (SPI-01 to SPI-04) inhibited SNEP2 more effectively than SENP1. However, for the cleavage of SUMO-2 precursor, they displayed similar inhibitory effect toward SENP1 and SENP2. They further tested SUMOylated proteins in Hela cells with SPI-01 treatment and performed heat-shock experiments. The result showed that SPI-01 had the inhibition of intracellular SENPs. The nuclear magnetic resonance (NMR) and quantitative enzyme kinetic analysis implicated that the inhibition mechanism was mainly non-competitive manner. These findings provide the possibility of designing substrate specific SENPs inhibitors (Madu et al., 2013).

Another study designed a new class of non-covalent SENP1 inhibitors based on pyridone scaffold via virtual design strategy. When SUMO1-AMC acted as a substrate, compound 11 showed an IC50 of 22 μM (with 0.1% BSA) and 3.7 μM (with 0.01% CHAPS) against SENP1. Compound 12 was obtained by replacing methyl amide with nitrile group. Compound 12 showed a larger difference in IC50 values when measured with different buffer additives. Compound 13 was obtained through replacing the central ester with an amide bond based on compound 11. To investigate the substrate specificity of SENP1 inhibitors, they detected the inhibitory concentrations when SUMO1, SUMO2 and SUMO3 acted as substrates, respectively. Compound 12 showed no selectivity on different SUMO substrates. Compound 11 and 13 exhibited 10-fold and 3-fold inhibitory activities on SUMO1 than on SUMO2 and SUMO3, respectively (Lindenmann et al., 2020).

## Conclusion and future perspectives

In this review, we summarized the function and mechanism of SENP1 in tumor proliferation, apoptosis, invasion, metastasis, stemness, angiogenesis, metabolism and drug resistance. Studies demonstrated that SENP1 was significantly overexpressed in many malignant tumors. Moreover, SENP1 acts as a promotive role in the occurrence and development of cancers through the deSUMOylation of target proteins. Previous researches have found that there is an interaction between ubiquitination and SUMOylation, which is critical for protein stability and activity. However, it is still unclear whether SUMOylation and other post-translational modifications exist similar interactions. The relation between SENP1-mediated deSUMOylation and protein translocation or distribution need more effort to investigate. In terms of angiogenesis, several studies have demonstrated the significant role of SENP1 in blood vessel growth and new neovascularization. However, the direct function of SENP1 in tumor angiogenesis has not been elucidated. These might be important directions for future research in cancer treatment targeting angiogenesis. Studies showed that SENP1 could be used as a novel prognostic marker and a potential therapeutic target for cancers. Thus, we should be committed to understand the action mechanism of SENP1 in cancers, which will help the development of antitumor drugs targeting SENP1. In recent years, many SENP1 inhibitors have been identified, designed and synthesized. Most of them have no entered clinical trials. SENP1 inhibitors are mainly natural products and synthetic compounds. Their species and quantity are very rich and have broad application prospects. Meanwhile, natural inhibitors have advantages of high activity, low toxicity, wide plant sources and low price as anti-cancer drugs. However, the application of SENP1 natural inhibitors faces many challenges and difficulties. For example, they have disadvantages such as poor water solubility, fast metabolism and low bioavailability. These defects limit them from becoming practical clinical drugs. Therefore, how to improve drug property through rational structural modification is a crucial problem. To date, many small molecule SENP1 inhibitors have been designed and synthesized based on the structure of natural products. Most of them are obtained by computational analysis and screening of compound libraries. Furthermore, the inhibitory potency of them is usually detected by molecular docking, molecular dynamics simulation or examination *in vitro*. However, they lack validation in cell experiments and animal models, which is a long way from practical application. In summary, directly targeting SENP1 promises to be a constructive anticancer strategy. Deeply exploring the action mechanism of its inhibitors and optimizing drug efficacy will provide more effective treatment options for tumorigenesis. Developing effective inhibitors targeting SENP1 and applying them to clinical practice are directions for future efforts in cancer therapy.
